# Elevated responses to constant facial emotions in different faces in the human amygdala: an fMRI study of facial identity and expression

**DOI:** 10.1186/1471-2202-5-45

**Published:** 2004-11-17

**Authors:** Jan Gläscher, Oliver Tüscher, Cornelius Weiller, Christian Büchel

**Affiliations:** 1 Neuroimage Nord, Department of Neurology, University Hospital Hamburg-Eppendorf, Martinistrasse 52, 20246 Hamburg, Germany; 2 Functional Neuroimaging Laboratory, Department of Psychiatry, Weill Medical School of Cornell University, 1300 York Ave, New York, NY, 10021, USA

## Abstract

**Background:**

Human faces provide important signals in social interactions by inferring two main types of information, individual identity and emotional expression. The ability to readily assess both, the variability and consistency among emotional expressions in different individuals, is central to one's own interpretation of the imminent environment. A factorial design was used to systematically test the interaction of either constant or variable emotional expressions with constant or variable facial identities in areas involved in face processing using functional magnetic resonance imaging.

**Results:**

Previous studies suggest a predominant role of the amygdala in the assessment of emotional variability. Here we extend this view by showing that this structure activated to faces with changing identities that display constant emotional expressions. Within this condition, amygdala activation was dependent on the type and intensity of displayed emotion, with significant responses to fearful expressions and, to a lesser extent so to neutral and happy expressions. In contrast, the lateral fusiform gyrus showed a binary pattern of increased activation to changing stimulus features while it was also differentially responsive to the intensity of displayed emotion when processing different facial identities.

**Conclusions:**

These results suggest that the amygdala might serve to detect constant facial emotions in different individuals, complementing its established role for detecting emotional variability.

## Background

Facial expressions and facial identities are important cues for the evaluation of social contexts [1,2]. Two main types of information have to be processed while seeing other persons' faces: a face has to be identified as belonging to a unique individual, establishing facial identity, while facial expressions have to be interpreted for emotional context, which is crucial for the social interaction [3]. Facial expression itself conveys two levels of information: first, facial emotions of others signal information about the emotional state and the benevolence or hostility of that person towards oneself; second, they convey information about the person's evaluation of the environment. The same emotional expression on several other persons' faces often signals a high degree of consistency in their evaluation of the current environment; they are therefore a particularly valid cue for one's own situational appraisal of this environment. In particular, the existence of specialized brain systems for the perception of fear expressions as a form of threat related to physical attack [4], points to the importance the brain attaches to social signals of potential environmental threats [5,6].

Visual analysis of human faces has been suggested to be achieved by a core system comprising the fusiform gyrus together with the inferior occipital gyrus, the superior temporal sulcus, and the amygdala [7,8]. The amygdala in particular has been shown to play a pivotal role in the processing and recognition of emotional facial expression, especially fear [9,10]. Since early functional imaging studies [11,12] showed amygdala activation to fearful face stimuli, a variety of imaging studies have led to a more detailed understanding of amygdala function in facial expression. Temporal properties of amygdala responses to stimulus repetition (i.e. habituation, e.g. [11,13-15]), the influence of attentional state [16] and awareness (e.g. [17-19]), as well as the amygdala's response to different types of emotional expression (most recently e.g. [20]) have been explored. Despite many studies investigating facial affect, only few have addressed the interaction of facial affect and identity. Preceding experiments on face processing were not explicitly designed to investigate differences in processing of facial identity and facial expressions, rather they examined the effects of changes in appearance, viewing angle, selective features and physical stimulus properties on face processing (e.g. [14,21,22]).

Recent advances to assess the effects of facial identity and emotional expression include one report on increased left amygdala activation to blocks of multiple novel vs. single identical faces displaying neutral or emotionless expressions [23]. Animated emotional expressions or identities, morphing either from neutral to emotional expression or from one neutral face to another, have been shown to elicit stronger activations in bilateral amygdala than static displays of these same stimuli [24]. Notably, these studies were not designed to investigate the interaction of identity and emotional expression as identity was only varied within the neutral expression condition. Most recently, Winston and colleagues have employed a factorial design to explore fMRI-adaptation to repeated presentations of two facial expressions in the context of an event-related fMRI experiment [25]. In contrast to earlier findings (e.g. [11,12]), the authors did not observe any significant signal changes in the amygdala in the context of their factorial design, suggesting that sparse stimulus presentations interleaved with a checkerboard might not be potent enough to evoke a task-related fMRI activation in this region.

**Figure 1:**
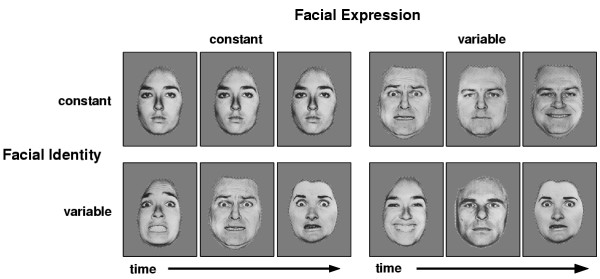
**Schematic display of the experimental design **We used a 2 × 2 (facial identity × facial expression) factorial design with either constant/variable identity or constant/variable expression as factor levels. Each cell represents one condition in the experiment. Facial identity varied between 4 faces (2 male, 2 female), expressions varied between 5 facial emotions ranging from maximally fearful over neutral to maximally happy with moderately fearful and happy in between. Pictures were presented sequentially at 1 Hz (900 ms stimulus duration, 100 ms blank screen).

Variability and constancy of identity and emotional expression are concepts that require stimulus integration over time, thus a sufficient number of stimulus presentations might be critical for a brain region such as the amygdala in order to detect stability or changes in the stimulus sequence. Therefore, we tested this interaction in a 2 × 2 factorial design (see Figure 1), with either constant or variable facial identity (factor 1), and either constant or variable emotional expression (factor 2), in a block design fMRI study. The conditions used were: (a) constant identity, constant expression (C_I_C_E_), (b) variable identity, constant expression (V_I_C_E_), (c) constant identity, variable expression (C_I_V_E_), (d) variable identity, variable expression (V_I_V_E_). To control for attentional effects during the procedure, an oddball task was included to avoid confounds by an emotional judgment or a gender differentiation task (the latter being a property of facial identity, [26]).

Given that numerous reports demonstrate amygdala activation in the processing of sequential facial expressions [11,12,27-29] and given the importance of different facial identities as a valid cue for making social inferences (see above), we hypothesized that constant emotional expressions displayed in different faces would elicit strong responses in the amygdala despite its well-described habituation to repeated stimuli [13,15,23]. Furthermore, we expected that constant *fearful *expressions (shown in different identities) would elicit the strongest amygdala activation given the evidence from lesion and imaging studies that this expression is a particularly potent activator of the amygdala [11,12,17,30,31]. Because we expected to find a stimulus repetition effect in the fusiform gyrus [14,32] we systematically examined responses to changing versus constant stimulus features, i.e., facial expression and identity in this area.

## Results

Analysis of the behavioral data showed that subjects maintained a high degree of task attention (98.03 % hits to oddball stimuli (facial stimuli at reduced luminance)). There was a trend of a main effect of identity in the reaction times (RTs) to the oddball stimuli (F_1,10 _= 4.859, p < 0.06), with longer RTs in the conditions of changing identities, but no significant effects of emotion or an interaction (emotion: F_1,10 _= 0.755, p > 0.40; interaction: F_1,10 _= 1.656, p > 0.20).

For the analysis of the imaging data we chose a statistical threshold of p < 0.05, corrected for a reduced search volume of interest for regions with a priori hypotheses (amygdala, fusiform gyrus). For other brain areas the threshold was set to a threshold of p < 0.05, corrected for the entire brain. Post-hoc contrasts of specific experimental conditions for detailed characterization of the experimental effects were carried out at an uncorrected significance threshold.

**Figure 2:**
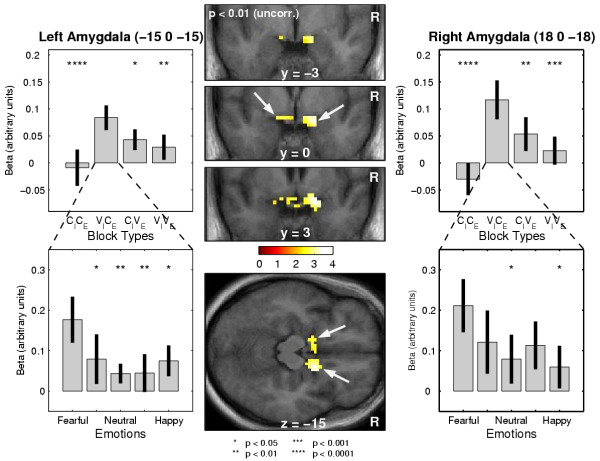
**Responses to different experimental conditions in both amygdalae **Coronal and transversal views of the activation foci in both amygdalae. These SPMs are derived from the interaction contrast identity × emotion (see Methods) and are superimposed on an averaged T_1_-weighted MR image of all our subjects. Arrows are pointing to the maximally activated voxel in the region. We report left amygdala activation in the light of bilateral activation shown previously in similar tasks [11,14]. **Top left and right panels. **Group mean parameter estimates (regression coefficients ± s.e.m.) to each of the experimental conditions for bilateral amygdala are shown. Condition V_I_C_E _elicits the largest activation of the peak voxels in both amygdalae and is significantly larger than all other experimental conditions. Asterisks (*) indicate significant difference of respective condition compared with condition V_I_C_E _at the specified uncorrected significance level. **Bottom left and right panels. **Detailed (emotion-specific) analysis of condition V_I_C_E_. Category labels refer to maximally fearful, neutral, and maximally happy facial expression. Intermediate categories are minimally fearful and minimally happy facial expression. Largest activations are elicited by maximally fearful faces. Asterisks (*) indicate significant differences between the respective emotion compared with maximally fearful faces at the specified uncorrected significance level.

In the amygdala we expected to find higher responses to constant emotional expressions in different facial identities. Thus in our 2 × 2 factorial design, this can be formally tested by an interaction. Data for all experimental conditions in the peak activation voxels found in our masked interaction contrast (thresholded at p < 0.01, for the purpose of visualization) are shown in the top left and right panels of Figure 2 with the corresponding significant voxels in the SPMs (arrows in middle panels). Applying a reduced search volume corresponding to an anatomical mask of the amygdala (see Methods) to these activations we found a highly significant peak in the right and another peak approaching significance in the left amygdala. We report the latter peak in light of bilateral amygdala activation in similar tasks shown previously [11,14]. Locations and statistics for this analysis for each peak are displayed in Table 1.

Post-hoc contrasts of the experimental conditions revealed that amygdala responses to V_I_C_E _were only significantly more active than responses to C_I_C_E _(left amygdala (-15 0 -15): T = 3.75, p < 10^-4^; right amygdala (18 0 -18): T = 4.76, p < 10^-4^), and also more active than the other two experimental conditions (C_I_V_E _and V_I_V_E_): left amygdala (-15 0 -15): V_I_C_E _vs. C_I_V_E_: T = 1.77, p < 0.05; V_I_C_E _vs. V_I_V_E_: T = 2.40, p < 0.01; right amygdala (18 0 -18): V_I_C_E _vs. C_I_V_E_: T = 2.19, p < 0.01; V_I_C_E _vs. V_I_V_E_: T = 3.30, p < 0.001.

In a further post-hoc analysis of the response to V_I_C_E _we decomposed this condition with respect to different emotional expressions. This analysis revealed that the V_I_C_E _activation is primarily caused by the response to maximally fearful expressions and (to a lesser degree) to other emotional expressions, but only marginal to neutral expressions (Figure 2, bottom graphs). Post-hoc pair-wise comparisons between maximally fearful and the other emotional expressions exceeded statistical thresholding (left amygdala (-15 0 -15): Fear Max vs. Fear Min: T = 1.95, p < 0.05, Fear Max vs. Neutral: T = 2.45, p < 0.01, Fear Max vs. Happy Min: T = 2.53, p < 0.01, Fear Max vs. Happy Max: T = 1.91, p < 0.05; right amygdala (18 0 -18): Fear Max vs. Neutral: T = 1.85, p < 0.05, Fear Max vs. Happy Max: T = 2.17, p < 0.05).

**Figure 3:**
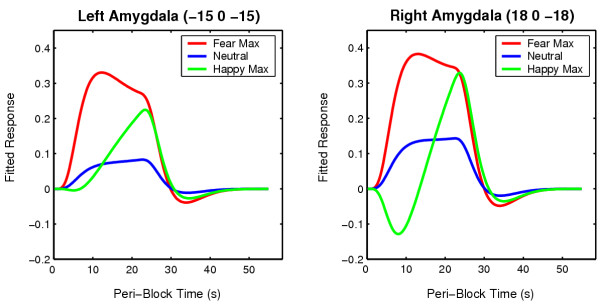
**Fitted time curves of BOLD-responses of the emotion-specific analysis of condition V_I_C_E _**Category labels refer to maximally fearful, neutral, and maximally happy facial expression, respectively. The fitted time courses from the peak voxels of SPMs derived from the interaction contrast identity × emotion (see Methods) are presented.

In order to characterize the time-course of the amygdala responses to the emotion-specific responses in condition V_I_C_E _we calculated the fitted responses for the most intense and the neutral expressions by multiplying the parameter estimates (regression coefficients) for the respective boxcar and exponential decay regressor with the canonical response function created by SPM during design specification. Figure 3 shows the time course for each emotion-specific response for each peak voxel in both amygdalae. While the overall height of the fitted responses parallel the parameter estimates of the boxcar regressors in Figure 3, we found a trend toward within-block habituation to fearful expressions, and an increase in the fitted responses for happy expressions (especially in the right amygdala) while neutral expression maintained the level of activation across the entire block.

**Figure 4:**
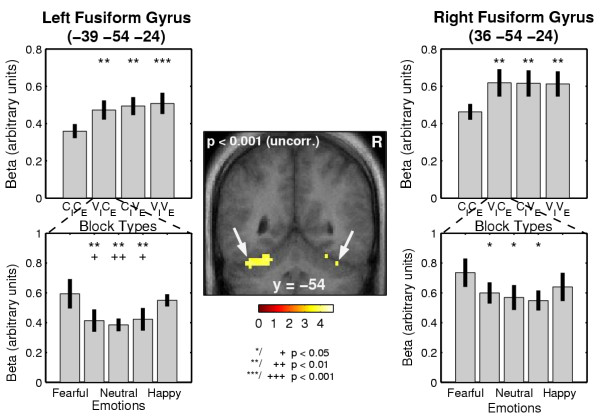
**Responses to different experimental conditions in left and right lateral fusiform gyri Middle panels. **Coronal view of the activation foci in the lateral fusiform gyri bilaterally. These SPMs are derived from the contrast that tested for a simple effect of changing stimulus features (see Methods) and are superimposed on an averaged T_1_-weighted MR image of all our subjects. Arrows are pointing to the maximally activated voxels in the region. **Left and right panels. **Group mean parameter estimates (regression coefficients ± s.e.m.) to each of the experimental conditions for bilateral lateral fusiform gyrus are shown. All conditions with changing stimulus features (identity, emotion; V_I_C_E_, C_I_V_E_, V_I_V_E_) elicit significantly larger activation of the peak voxels in both fusiform gyri than condition C_I_C_E _in which the same pictures were shown for the entire block. Asterisks (*) indicate significant differences of respective condition compared with condition C_I_C_E _at the specified uncorrected significance level. **Bottom left and right panels. **Detailed (emotion-specific) analysis of condition V_I_C_E_. Category labels refer to maximally fearful, neutral, and maximally happy facial expression. Intermediate categories are minimally fearful and minimally happy facial expression. Largest activations are elicited by maximally fearful faces. Asterisks (*) indicate significant differences between the respective emotion compared with maximally fearful faces at the specified significance level, pluses (+) indicate significant differences between the respective emotion compared with maximally happy faces at the specified uncorrected significance level.

Based on previous work [22,32] we expected activation in the fusiform gyrus to be dependent on stimulus changes irrespective of whether emotional expression, facial identity, or both were varied. We also expected to detect an influence of emotion type and intensity on fusiform activity [12,22]. Accordingly, we compared conditions with at least one changing stimulus feature (identity, emotion) to the condition in which the same picture was repeated for the entire block. Here we found evidence for an effect of changing stimulus features. Figure 4 depicts significantly activated voxels in the lateral fusiform gyrus for this contrast (thresholded at p < 0.001 for the purpose of visualization), with arrows pointing to the voxels of peak activation. Location and statistics corrected for a reduced search volume for this region reported by Vuilleumier and colleagues [33]; see Methods) are shown in Table 1. These locations correspond well to previously reported regions linked to human face processing [34].

Pair-wise post-hoc contrasts of the four experimental conditions revealed that the lateral fusiform gyrus was significantly more activated by all experimental conditions with at least one changing stimulus feature (V_I_C_E_, C_I_V_E_, V_I_V_E_) than by the condition with repeated presentation of the same picture (C_I_C_E_): left lateral fusiform gyrus (-39 -54 -24): V_I_C_E _vs. C_I_C_E_: T = 2.75 p < 0.01, C_I_V_E _vs. C_I_C_E_: T = 3.09, p < 0.01, V_I_V_E _vs. C_I_C_E_: T = 3.77, p < 0.001; right lateral fusiform gyrus (36 -54 -24): V_I_C_E _vs. C_I_C_E_: T = 2.98, p < 0.01, C_I_V_E _vs. C_I_C_E_: T = 2.79, p < 0.01, V_I_V_E _vs. C_I_C_E_: T = 3.01, p < 0.01.

A further post-hoc analysis of the condition V_I_C_E _revealed an effect of emotion intensity with the maximal intensity expressions eliciting stronger activations than those with reduced intensities and the neutral expression. Pair-wise contrasts of the emotion-specific V_I_C_E _condition revealed that maximally fearful expressions elicited significantly larger activation than neutral and emotional expressions of minimal intensity (left lateral fusiform gyrus (-39 -54 -24): Fear Max vs. Fear Min: T = 2.6, p < 0.01; Fear Max vs. Neutral: T = 2.79, p < 0.001; Fear Max vs. Happy Min: T = 2.38, p < 0.01; right lateral fusiform gyrus (36 -54 -24): Fear Max vs. Fear Min: T = 1.72, p < 0.05; Fear Max vs. Neutral: T = 1.94, p < 0.05; Fear Max vs. Happy Min: T = 2.27, p < 0.01; indicated by asterisks (*) in the bottom panels of Figure 4). However, only in the left lateral fusiform gyrus the same post-hoc tests were significant when contrasting maximally happy expressions with all others (Happy Max vs. Happy Min: T = 1.84, p < 0.05; Happy Max vs. Neutral: T = 2.54, p < 0.01; Happy Max vs. Fear Min: T = 1.99, p < 0.05, indicated by pluses (+) in the bottom panels of Figure 4).

## Discussion

We investigated the interaction of facial identity and expression in 2 × 2 factorial blocked fMRI study which uniquely enabled us to compare all combinations of facial identity and expression within the same experiment, therefore the results of the present study complement and extend the results of previous studies on facial identity and expression processing in the human brain. In support for our hypotheses we found distinct response patterns in bilateral amygdala and bilateral lateral fusiform gyrus. While the lateral fusiform gyri can be characterized by a binary response pattern corresponding to an effect of changing stimulus feature, the amygdala responded maximally to constant emotional facial expressions in combination with changing facial identity. In addition, in the V_I_C_E _experimental condition we found an effect of emotion intensity in the fusiform gyrus, with stronger activations to maximally intense expression irrespective of valence when compared with neutral or modestly intense expressions. While we found a (non-significant) trend for the same modulation of the V_I_C_E _activation by emotion intensity in the left amygdala the general pattern when including the right amygdala rather conforms to a specific sensitivity for maximally fearful expression which elicited the strongest activation.

### Fusiform gyrus activations

The binary response pattern conforming to sensitivity for changes in stimulus features in the lateral fusiform gyrus might be explained by reference to the proposed network of face processing in humans [7]. Haxby and colleagues hypothesize that this part of the distributed network is especially sensitive to facial stimulus configurations (and the variability of these configurations). Evidence supporting this conclusion has been reported in a study by Vuilleumier and colleagues who demonstrated a significant repetition suppression effect in the fusiform gyrus in response to the second compared to the first presentation of a given facial identity [22]. Similarly, Rotshtein and colleagues demonstrated stronger activation in face related voxels in the lateral occipital complex (LOC) for blocks with different identities (resembling the condition V_I_C_E _of the present study) compared to blocks of the repetition of the same identity (our condition C_I_C_E_) [14]. Another study investigating the influence of facial expression and identity on fusiform activation found reduced activation to facial identity but not to expression and no interaction of these two factors [25]. Our data support and extend these findings with respect to identity in the fusiform gyrus, as we found evidence for an effect of changing stimulus features in the lateral fusiform gyrus irrespective of the dimension of variation (facial identity, emotional expression collapsed over all emotions, or both).

Additionally, we found an effect of emotion intensity in the fusiform gyrus activity in condition V_I_C_E _with higher activation to maximally fearful and happy expressions relative to with neutral expressions, paralleling earlier findings which report stronger activation to fearful than to neutral expressions [22,24,27,33,35]. Rotshtein and colleagues show significantly larger activation for aversive versus happy expressions in repeated presentations (C_I_C_E_), suggesting an identity repetition × emotion interaction [14], while others fail to find an effect of negative (or positive) expression in the fusiform gyrus [25]. This apparent negativity bias might be a confound of stimulus selection as researchers use negative material more frequently than positive stimuli. In fact, the underlying mechanism might be an enhanced attentional processing of arousing facial expressions [36], which are usually also the most negative faces. Our finding of an effect of emotion intensity supports this notion more convincingly as we also show increased activation in the fusiform gyrus to happy expressions. An underlying arousal dimension that exerts a modulatory effect on the activation in this region might also explain the lack of an effect of expression in the findings of Winston and colleagues [25], as they did not include a neutral (low arousal) expression in their stimulus set.

Two recent studies suggest that the low frequency information of facial stimuli might drive the modulatory effect of arousal, which is thought to be a feedback influence of the amygdala, which also displays a preference for low frequency information [22,35].

### Amygdala activations

Our 2 × 2 factorial blocked fMRI design allowed us to compare between the possible conditions of constant and variable facial identity and expression. We show for the first time a maximum of activation of the amygdala to variable facial identities displaying the same emotion (V_I_C_E_) compared to all three other main conditions (C_I_C_E_, C_I_V_E _and V_I_V_E_). Furthermore, within this condition (V_I_C_E_) maximally fearful expressions elicited the strongest amygdala activity. The result of the present study replicates and extends, in part, the earlier findings that also utilized blocked presentations of facial expression with stimulus configurations resembling one or more of our experimental conditions (V_I_C_E _blocks: [11,12,28,37,38]; C_I_C_E _and V_I_C_E _blocks: [14]; V_I_C_E _and V_I_V_E _blocks: [39]). In light of our findings, the significant amygdala activation to fearful expression in the aforementioned studies might have been obtained because they employed a stimulus presentation conforming to our condition V_I_C_E_. Interestingly, in a study comparing dynamically morphed and static presentations of facial stimuli, LaBar and colleagues [24] also report amygdala activation when neutral faces are morphed into each other (a dynamic version of our condition V_I_C_E_) as compared to static and repeated presentation of the same facial stimuli (the condition C_I_C_E _in the present study). A closer inspection of our findings in Figure 2 reveals a similar trend, as the neutral expression in the V_I_C_E _condition still elicited a larger signal change than the condition C_I_C_E_. Thus, our comprehensive factorial design can relate these earlier findings in the broader context of constancy and variability of identity and expression.

Many imaging and lesion studies have documented activations or behavioral impairments following the presentation of fear-related stimuli (for review see [9]). Our decomposition of the amygdala activation in condition V_I_C_E _supports this claim. However, recent studies, which used different sensory modalities and carefully controlled for the often confounded dimensions of valence and intensity, argued for an effect of emotion intensity (arousal) in the amygdala [40-42]. Further support for this interpretation comes from a comprehensive study investigating the effects of different expressions of basic emotions during direct and incidental stimulus processing [20]. The authors found no specific effect for a particular emotional expression; they rather report amygdala activations when comparing high vs. low intensity exemplars of the facial stimuli. Although we also find trends for an effect of emotion intensity (Figure 2, bottom left panel), the question of specific amygdalar fear sensitivity or a more general arousal sensitivity remains equivocal based on our findings.

Interestingly, Winston and colleagues [25], employing a similar design as in the present study, failed to observe significant signal changes in the amygdala. The divergent findings might be explained by the difference between our blocked and their event-related design, in which they sequentially presented pairs of facial stimuli. Sparse stimulus presentations in that study might not be sufficient to trigger activation in the amygdala, as this structure might decode a more sustained stimulus train for assessing the biological relevance of the current situation (see below). Furthermore, in their study, the pairs of faces presented were separated by an face-outlined checkerboard [25] which might have prevented the induction of an emotional state that would be encoded by the amygdala.

The temporal properties of amygdala responses to facial expressions are crucial. Many studies show habituation of the amygdala response to blocks of directly adjacent fearful vs. neutral or happy expressions with constant identities over the course of 30 to 80 sec [13,15,38]. This is comparable to the C_I_C_E _condition in the present study in terms of presented stimuli but not with respect to block length and block order. Interestingly, when the C_I_C_E _condition in the present study is analyzed for the comparison of maximum fear vs. neutral blocks a non-significant trend for left ventro-lateral amygdala activity (data not shown) is seen, indirectly supporting those findings.

Other studies used fearful and neutral V_I_C_E _conditions showing within-block and across-block habituation with fixed alternating block order [11] or in the comparison of V_I_C_E _vs. C_I_C_E _blocks [14,23] in designs with pseudo-randomized block order. Likewise, we also show a highly significant difference between the V_I_C_E _and C_I_C_E _conditions in the amygdala. The response profile of the amygdala for maximal fearful expressions in the V_I_C_E _condition trends toward a within-block habituation effect (Figure 3). In contrast, the response profile for neutral expressions exhibits a sustained response during the entire block albeit at a much lower overall level. This aspect parallels findings by Breiter and colleagues [11], although direct comparison is limited because that study showed habituation on a larger timescale (between blocks). The within-block increase of amygdala activation to happy expressions, however, suggests that the temporal nature of signal changes in the amygdala might be more complex. This delayed onset of activation to happy faces is in accordance with an evolutionary interpretation of our findings. The amygdala is located in a critical position on the efferent pathway that is involved in the preparation of autonomic responses to threatening situations [43]. Because happy expressions are usually a valid signal for non-threatening situations that do not require fight or flight responses, amygdala activation is not needed at an early stage. The explanation also holds for the early but overall attenuated response to neutral expressions that are inherently ambiguous, which thus prompt for sustained perceptual processing and, potentially, for preparation of defensive behavior [44]. Additionally, Wright and colleagues [23] showed greater amygdala activation to neutral V_I_C_E _blocks compared with neutral C_I_C_E _blocks. Clearly, further research is needed to characterize the temporal evolution of amygdala activation during this type of blocked stimulus presentation in more detail.

One might argue that the responses in the conditions with variable emotional expression (C_I_V_E_, V_I_V_E_) were reduced because within those blocks variable emotional and neutral expressions were intermixed, possibly leading to smaller signal changes within those blocks. However, subjects were presented with the same number of stimuli of each emotional expression in every experimental condition. Therefore, differences cannot be attributed to a varying number of emotional expressions seen in different conditions. Thus, if the conditions with variable emotions elicit a smaller signal change because of the neutral faces within them, this effect should also apply to the blocks of constant neutral expressions in different faces (V_I_C_E _condition). This effect is in fact shown in the lower bar graphs in Figure 2, especially in comparison to the other emotional V_I_C_E _conditions. Because we found an overall elevated response in the amygdala to the condition with constant emotions in changing identities (V_I_C_E_) compared to conditions with variable emotions (C_I_V_E_, V_I_V_E_; see Figure 2 upper bar graphs), we argue that the observed differences between our experimental conditions represent a true effect of the sequential stimulus configuration within this experimental design. Although we can not ultimately exclude the possibility of confounding order effects within the categories of variable emotion conditions (C_I_V_E_, V_I_V_E_), this does not diminish the main point of this study, namely that the human amygdala is most responsive to the sequential presentation of faces with constant emotion (fearful) and varying identity. To further confirm and generalize this finding future studies are needed using additional emotions (e.g. anger, sadness, etc.).

Novelty effects of changing facial identity and stimulus order of the different conditions might also be claimed as an explanation for the strong responses to the condition V_I_C_E _[23]. But in contrast to the latter study, subjects in our study were familiarized with the stimuli before the experiment and the same facial identities were presented repeatedly throughout the experiment, thus minimizing stimulus novelty. Furthermore, if novelty detection were the main factor driving the amygdala response, the condition V_I_V_E _should have also elicited a strong amygdala response which it did not (see Figure 2). There was also no systematic sequence of blocks of constant facial identities followed by variable facial identities in the present study which has been shown to relatively increase the activation to the multiple identity condition presented secondly [23].

It is interesting to note that the peak activation within the amygdala is located in the medio-dorsal part of this structure. Previous imaging studies of the processing of facial affect have predominantly reported their activations in the dorsal amygdala (for review see [10], Figure 3). The dorsal amygdala has also been associated with the representation of ambiguous stimuli, such as fearful expressions that do not signal a potential threat directly [10,29,44]. However, given the resolution and post scan smoothing of the functional images in this and other functional imaging studies, the localization of amygdala activity should be discussed with caution.

### The potential biological relevance of constant facial emotion

Sensitivity for constant facial emotions in different identities can be seen as a process that integrates stimuli with respect to the conveyed emotion over a certain amount of time. Thus, environmental stimuli (such as facial expressions) are compared to each other to detect changes to stability in the emotion, a concept that can be described as *emotion constancy*. In this context, constant facial emotions in different individuals signal a high degree of consistency in others' appraisals of the environment, and constitute a more valid cue for one's own appraisal.

Furthermore, facial expressions differ in their degree of saliency which often reflects the biological relevance of the stimulus that evoked the expression [18,44]. For example, a fearful face as a reaction to a threatening stimulus is more salient and calls more immediately for appropriate action than a neutral facial expression. We found elevated response in the amygdala especially to these salient (fearful) expressions because these constant salient facial emotions call for an immediate situational appraisal and subsequent action.

Our findings gain support and extend the findings of earlier imaging studies on the processing of human facial emotions which employed an experimental design similar to our condition V_I_C_E _and found significant activation in the left dorsal amygdala with constant fearful expressions [11,12]. Taken together, these results, as well as our own findings in this experiment, suggest that the same emotional expressions displayed in many different faces are potent stimuli that activate the amygdala and might serve the detection of emotion constancy. Conceptually, conditions with variable emotionality (C_I_V_E_, V_I_V_E_) can be seen as noise in the context of the detection of constant emotions among others and thus, it is not surprising that the amygdala shows less signal change in these conditions.

The perception of emotional facial expressions in others yields insights into their evaluations of the environment and guides one's own emotional and behavioral reactions. Encountering the same emotional expression in many different people (emotion constancy) is an especially valid and readily available cue for making subsequent inferences about the potential harmfulness of an environmental situation. These inferences would have direct implications for evolutionary survival and must have been a central feature of human ancestral cognitive abilities [45]. They also remain essential for safe locomotion in our current complex environment as they reduce the time spent in a potentially threatening situation, because the perceiving subject can avoid energy-intensive and time-consuming search for potential threats in the environment [46]. For example, encountering the same fearful expression in several different people (emotion constancy) strongly implies an activation of the fear system [45,47] and that precaution and avoidance behavior are adequate reactions in this situation. Our data, in particular the large response to constant maximally fearful expressions in different individuals, suggest that the amygdala plays a central role in the neurobiological realization of this environmental evaluation.

## Conclusions

Emotional facial expressions are an important cue for the appraisal of an environmental situation. Our study has demonstrated a new perspective on the functional characterization of the amygdala involved in the perceptual processing of human faces by incorporating the dimensions of constancy and variability detection in these stimuli. These are essential for the assessment of the temporal dynamics of social situations.

## Methods

### Experimental design

A 2 × 2 factorial design (identity × emotional expression) across 4 different block types was used: (a) constant identity, constant expression (C_I_C_E_), (b) variable identity, constant expression (V_I_C_E_), (c) constant identity, variable expression (C_I_V_E_), (d) variable identity, variable expression (V_I_V_E_). Figure 1 schematically displays our experimental design showing three consecutive stimuli from one block within each cell of the factor table.

### Subjects

13 Subjects (8 females, 10 right-handed) participated in this fMRI study. The mean age was 25.6 (SD 7.8). The data sets of two subjects were excluded from further image analysis because of radio frequency artifacts caused by the scanner leaving a total of 11. All subjects were fully informed about the experimental procedure and signed a consent statement which was approved by the local ethics committee.

### Experimental procedure

The procedure was completed in one imaging session with 4 runs each containing 20 blocks of stimulus presentation. Within one run the blocks were interleaved with 15 s of central fixation (rest period). Each run started with a rest period of 20 s. 20 stimuli were presented per block at a rate of 1 Hz. Stimuli were shown for 900 ms interposed with a 100 ms gray blank of mean luminance to make transitions between stimuli less abrupt. Numbers and types of both facial expressions and facial identities were counterbalanced across the entire experiment. Conditions varied only in terms of the sequential configuration of the stimuli within the blocks. Stimulus order within each block was pseudo-randomized and fixed. The sequence of blocks within each run was counterbalanced, pseudo-randomized and fixed to minimize stimulus order effects. Additionally, the sequence of the four runs was pseudo-randomized across subjects assuring different orders of runs in each subject.

Stimuli consisted of 4 different faces (facial identities) drawn from the Ekman series of facial affect [48] with 5 different emotional expressions ranging from fearful to happy expressions, including neutral. Two intermediate expressions displayed fearful and happy emotions at reduced (50 %) intensities. Those face pictures were interpolations using computer morphing procedures [49] similar to those in other studies. Subjects were instructed to fixate on a small red fixation dot presented in the middle of the viewing monitor while simultaneously attending to the entire stimulus presentation.

In order to maintain and control for attentional effects during the procedure we included an oddball task as we were seeking to avoid confounds by an emotional judgment or a gender differentiation task (latter being a property of facial identity, [26]). As oddball stimulus we occasionally presented the facial stimuli with reduced luminance of the entire face while leaving the stimulus visible which was not expected to affect activation in high-order visual areas [50]. We chose to manipulate the entire facial stimulus for the oddball task in order to keep the subjects attention on the entire stimulus rather than on some small feature. Subjects were instructed to respond with a button press whenever an oddball target appeared. The number of oddball targets per block ranged from 2 to 4 to avoid subjects' expectancy effects. In order to prevent a systematic effect of the number of oddballs in the data analysis, the number of oddball stimuli was counterbalanced across blocks and conditions. Subjects were familiarized with the oddball task in a practice session prior to the first scanning run.

### Image acquisition

Imaging was performed on a 1.5 T Magnetom Vision (Siemens, Erlangen, Germany) scanner. 43 transversal slices of echo-planar (EPI) T2* weighted images in each volume with a slice thickness of 2 mm and 1 mm gap (TR = 3.5 s, TE = 40 ms, flip angle 90°, FoV 192 × 192 mm^2^, matrix 64 × 64) were acquired. A total of 204 volumes were collected per run.

### Image processing

Image processing and statistical analysis were carried out using SPM99 for the single subject analysis and SPM2 for the group analysis [] All volumes were realigned to the first volume, spatially normalized to a standard EPI template [51] using sinc interpolation and finally smoothed with a 11 mm isotropic full width at half maximum (FWHM) Gaussian filter to account for anatomical differences between subjects and to allow statistical inference using Gaussian Random Field theory.

### Statistical analysis

The data of 11 subjects were included in the statistical analysis. Data analysis was performed using the mass univariate general linear model as implemented in SPM99 and commenced by specifying the design matrix for each subject using a boxcar and an exponential decay regressor for modeling the hemodynamic response to each experimental condition. The boxcar regressor models the mean activation within the block while the exponential decay regressor (time constant 4 s) models decreases and increases (through negative contrast weights) within the block. The conditions with constant emotional expressions (C_I_C_E_, V_I_C_E_) allowed a decomposition into emotion-specific components. Thus each of these two conditions involved 10 regressors (boxcar and exponential decay for 5 different expression), while each of the other conditions (C_I_V_E_, V_I_V_E_) were specified with two regressors. Data were high-pass filtered at 1/120 Hz. Serial autocorrelation was controlled by superimposing a known autocorrelation in form of temporal smoothing using a low-pass filter at 4 sec filter width. Successively, contrasts for each experimental condition were computed by averaging the same block type across runs and multiplying the design matrix with the contrast vectors.

These single-subject contrast images were then taken to the second level oneway ANOVA [52,53] in SPM2 allowing for an appropriate non-sphericity correction [54]. This correction is equivalent to the Greenhouse-Geisser procedure in multivariate ANOVA analyses and allows for correct assessment of the error covariance matrix, hence securing valid inference in the group comparisons.

In order to detect voxels that show elevated responses to the same expression displayed in different individuals we constructed the interaction contrast of our 2 × 2 design. Hence, we created the contrast [(V_I_C_E _> C_I_C_E_) > (V_I_V_E _> C_I_V_E_), p < .01 for the purpose of visualization] for the amygdala and masked it with the contrast [(V_I_C_E _> C_I_V_E_), p < .05] to exclude regions showing higher activations to C_I_V_E _than to V_I_C_E_. For our hypothesis in the fusiform gyrus we used a contrast that compared conditions with at least one changing stimulus feature (facial identity, emotional expression, or both) with the condition in which the same stimulus was shown for the entire block [(V_I_C_E _+ C_I_V_E_+ V_I_V_E_) > 3 × C_I_C_E_].

T-statistics for the assessment of significant regional activation were assembled into Statistical Parametric Maps (SPMs) which refer to the probabilistic behavior of Gaussian random fields [55]. Our threshold was set at p < .05 (corrected). Because we had region-specific hypotheses for the amygdala and the lateral fusiform gyrus, we applied a reduced search volume to our amygdala activation which was derived by an anatomical mask created with MRIcro [56] on the template brain of the Montreal Neurological Institute (MNI, [57]). With additional visual reference to a high-resolution anatomical atlas [58] we outlined the amygdala on each slice of the MNI template brain. Thus, the amygdala search volume comprised 77 voxels, or 2071 mm^3 ^on the right side and 74 voxels, or 2005 mm^3 ^on the left side. Similarly, we applied a 10 mm radius sphere to our activation peaks in the lateral fusiform gyrus centered on coordinates reported by Vuilleumier and colleagues when contrasting faces vs. houses in a functional localizer task [33]; see Table 1). For additional brain areas not included in our volumes of interest we corrected for the entire brain volume.

For the emotion-specific analyses of condition V_I_C_E _(Figure 2 and 4, bottom panels) we referred to specific contrasts for each emotional expression created at the single-subject level. These contrast images were raised to another second level one-way ANOVA to test, for example, for significant activations to maximally fearful faces within this condition. These statistical comparisons were carried out at an uncorrected significance threshold.

## List of abbreviations

EPI: echo-planar imaging

fMRI: functional magnetic resonance imaging

LOC: lateral occipital complex

RT: reaction time

SPM: statistical parametric map

V_I_V_E_: variable identity, variable emotion

V_I_C_E_: variable identity, constant emotion

C_I_C_E_: constant identity, constant emotion

C_I_V_E_: constant identity. variable emotion

Fear/Happy Max: maximally fearful/happy expression

Fear/Happy Min: minimal fearful/happy expression.

## Authors' contributions

J.G. and O.T. designed, coordinated, and conducted data collection, analysis, and interpretation. C.B. and C.W. conceived of the study and participated in its design, analysis and interpretation. All authors read and approved the final manuscript.

## Figures and Tables

**Table 1:** Location of significant voxels in amygdala and lateral fusiform gyrus.

**Region**	**x**	**y**	**z**	**Z-value**
Left Amygdala	-15	0	-15	2.65
Right Amygdala	18	0	-18	3.55**
Left Fusiform Gyrus	-39	-54	-24	3.43*
Right Fusiform Gyrus	36	-54	-24	3.19*

Statistics in the amygdala are based on a reduced search volume based on an anatomical mask (see Methods). In the fusiform gyri we employed a search volume with a 10 mm radius sphere centered on coordinates reported by Vuilleumier and colleagues [33] which derived from a comparison between face and house stimuli in a functional localizer [± 45 -54 -21]. All reported activations are corrected for the volume of interest. MNI coordinates. * p < .05 (corrected); ** p < .01 (corrected).
